# Monitoring the Release of Methylglyoxal (MGO) from Honey and Honey-Based Formulations

**DOI:** 10.3390/molecules28062858

**Published:** 2023-03-22

**Authors:** Md Lokman Hossain, Lee Yong Lim, Katherine Hammer, Dhanushka Hettiarachchi, Cornelia Locher

**Affiliations:** 1 Division of Pharmacy, School of Allied Health, University of Western Australia, Crawley 6009, Australia; 2 School of Biomedical Sciences, University of Western Australia, Crawley 6009, Australia; 3 Cooperative Research Centre for Honey Bee Products Limited, 128 Yanchep Beach Road, Perth 6035, Australia

**Keywords:** release profile, methylglyoxal, honey-based formulation, Franz diffusion cell, High-Performance Liquid Chromatography

## Abstract

Methylglyoxal (MGO) is considered to be one of the vital components responsible for the anti-bacterial activity of *Leptospermum* spp. (Manuka) honey. While many studies have demonstrated a dose-dependent antibacterial activity for MGO in vitro, from a therapeutic viewpoint, it is also important to confirm its release from Manuka honey and also from Manuka honey-based formulations. This study is the first to report on the release profile of MGO from five commercial products containing Manuka honey using a Franz diffusion cell and High-Performance Liquid Chromatography (HPLC) analysis. The release of MGO expressed as percentage release of MGO content at baseline was monitored over a 12 h period and found to be 99.49 and 98.05% from an artificial honey matrix and NZ Manuka honey, respectively. For the investigated formulations, a time-dependent % MGO release between 85% and 97.18% was noted over the 12 h study period.

## 1. Introduction

Honey, a highly viscous natural substance, is produced by bees from the nectar of flowers (blossom honey) or the exudation of living parts of plants or insect excretions (honeydew honey) [1,2]. Worker bees collect nectar into their ‘honey stomach’ and break up disaccharides (primarily sucrose) into monosaccharides (i.e., glucose and fructose). Then, worker bees pass the digested nectar on to younger house bees, which take it into the colony and pack it away in hexagon-shaped cells made from beeswax. There, hive nectar is converted into honey by evaporation of some of its moisture content, which is achieved by the house bees’ fluttering of their wings over the nectar-filled cells [2,3]. Once concentrated enough to ensure no microbial spoilage during storage, the cells are capped using fresh beeswax. The chemical composition of honey has been studied in detail and more than 400 honey constituents have to date been identified [1,2,3]. About 80% of honey is made of sugars, followed by water (approximately 17%) and ‘other’ constituents (approximately 3%), which all play a critical role in honey’s different characteristics, including its various bioactivities [1,2,3,4].

**Figure 1:**
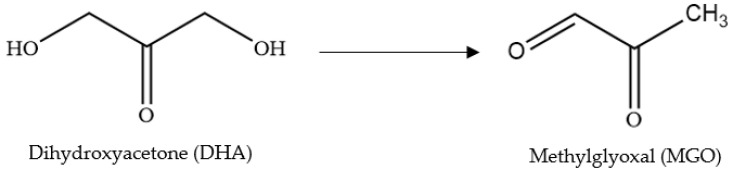
Formation of MGO from DHA.

Honey has been used in a variety of medicinal applications by numerous cultures for thousands of years due to its bioactivities, for example, its antibacterial, antioxidant, anticancer, antiparasitic, antiviral and antidiabetic effects [1,2,3]. The therapeutic potential of honey is related to its complex compound profile, including a range of phenolic compounds, organic acids, enzymes (e.g., diastase, glucose oxidase, and invertase), minerals (e.g., potassium, iron, zinc) and other minor constituents. Honey’s antibacterial effect is mainly influenced by its high osmolarity, acidity (low pH), enzymatic generation of hydrogen peroxide (H_2_O_2_) and nitric oxide (NO) on exposure to water and the presence of bee defensin-1 and other bee-related enzymes as well as other minor constituents (e.g., phenolics, flavonoids, organic acids, enzymes, minerals) [4,5,6,7,8]. In the case of so-called ‘peroxide honeys’, their antibacterial activity has been mainly associated with the generation of H_2_O_2_, whereas in so-called ‘non-peroxide honeys’ [9,10,11], activity is commonly related to the presence of methylglyoxal (MGO). Non-peroxide honeys are commonly derived from the *Leptospermum* species [12,13]. In these honeys, MGO is irreversibly formed during honey maturation, storage, and processing from the dehydration of a precursor molecule, dihydroxyacetone (DHA) (Figure 1), a compound found naturally at high levels in *Leptospermum* nectar and unripe honey [14].

The well-known bioactivities of honey have made it an interesting alternative medicine, which in turn, has motivated researchers to also formulate honey-based products, such as gels, dressings, or ointments, in particular for wound healing. Honey has been impregnated with other materials, for example, collagen, gelatin, starch, cellulose, alginate, or agarose to derive wound care products, which, compared to pure honey, might be more convenient to use and therefore more appealing to patients and health care professionals. Currently, there are a number of US Food and Drug Administration (FDA)-approved honey-loaded products commercially available [15]. Honeys sourced from the tree genus *Leptospermum* (native to Australia and New Zealand), which are commonly referred to as Manuka honeys, are frequently incorporated into these products [15,16]. They are mostly used for the treatment of wounds, minor abrasions, lacerations, minor cuts, minor scalds and burns, and diabetic foot ulcers [15,16,17].

The release of active ingredient(s) from the formulation matrix is often a critical product attribute in both medicinal product development and manufacture. The in vitro release profile can reveal core information on the dosage form and its behaviour, as well as provide details on the release mechanism and kinetics, enabling a rational and scientific approach to drug product development [18]. In a manufacturing context, in vitro drug release testing is used routinely in quality control to support batch release [18]. As a predictor of drug bioavailability and therapeutic outcomes, the in vitro release profile might be an indirect measure of the effectiveness of the formulation [18]. Similar to other pharmaceutical products, the therapeutic effects of honey-based medicinal products can also be expected to be potentially influenced by the release of appropriate active components (i.e., MGO, phenolics, flavonoids) from the honey-incorporated formulation matrix upon topical application.

The Franz diffusion cell is a simple and widely used analytical tool to evaluate the in vitro drug release from topical dosage forms such as gels, creams, and ointments [19,20,21]. The apparatus consists of a donor and receptor compartment between which the membrane is placed. The drug permeation rate from the donor compartment through the membrane into the receptor is determined by measuring the amount of drug released over time. A suitable analytical tool, for example, High Performance Liquid Chromatography (HPLC), is then used to determine the amount of drug permeation.

To date, however, the release profile of MGO from honey-based formulations, and also from pure honey, despite their popularity as medicinal agents, has not been investigated. In this study, using a Franz diffusion cell, the in vitro release of MGO from honey and from commercial honey-based products was therefore monitored by HPLC.

## 2. Results

**Figure 2:**
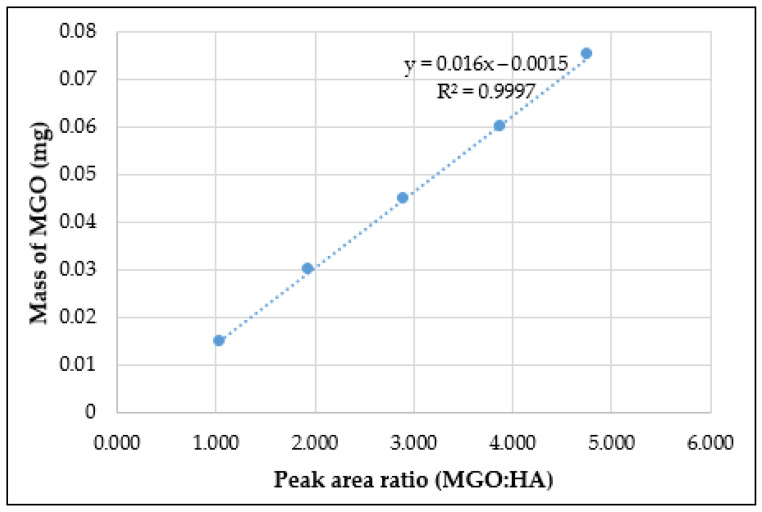
MGO calibration curve.

**Figure 3:**
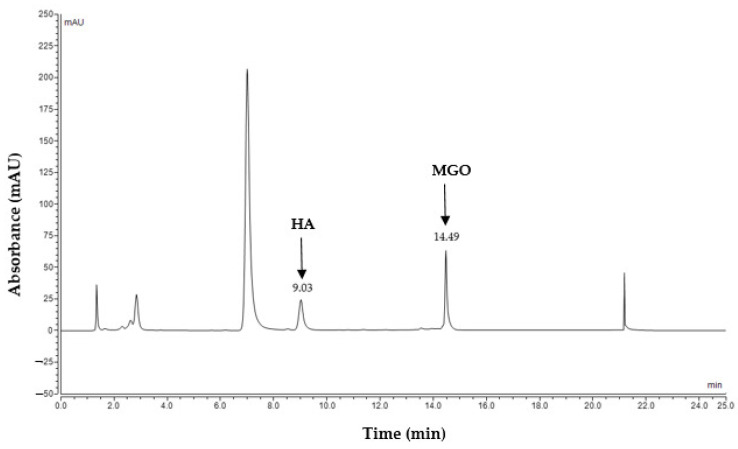
HPLC chromatogram of MGO standard (0.015 mg/mL) and HA internal standard (0.075 mg/mL) at λ = 263 nm.

A 5-point calibration curve (Figure 2) for the quantification was obtained by calculating the ratio of the peak area of MGO (RT 14.49 min) to the peak area of the HA internal standard (RT 9.03 min) in the chromatogram (Figure 3) [22]. The equation derived from the linear regression analysis of the calibration was used to quantify the baseline MGO and also the release profile of MGO from MGO-spiked artificial honey, pure Manuka honey and the five commercial formulations containing Manuka honey.

MGO:HA (peak area) = 0.0015 × mass of MGO (mg)

**Figure 4:**
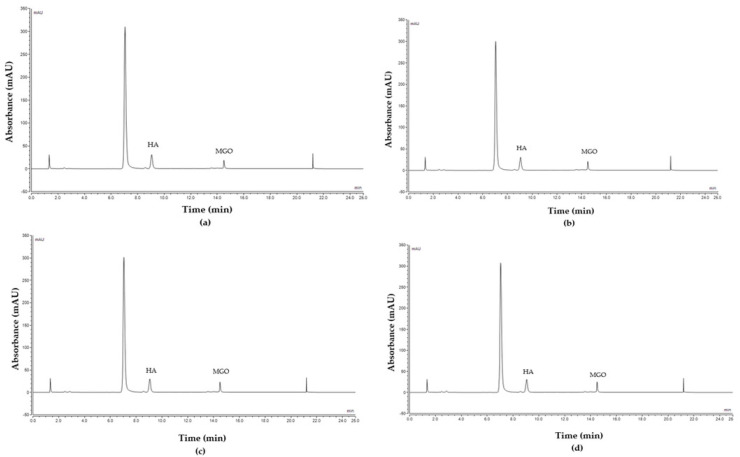

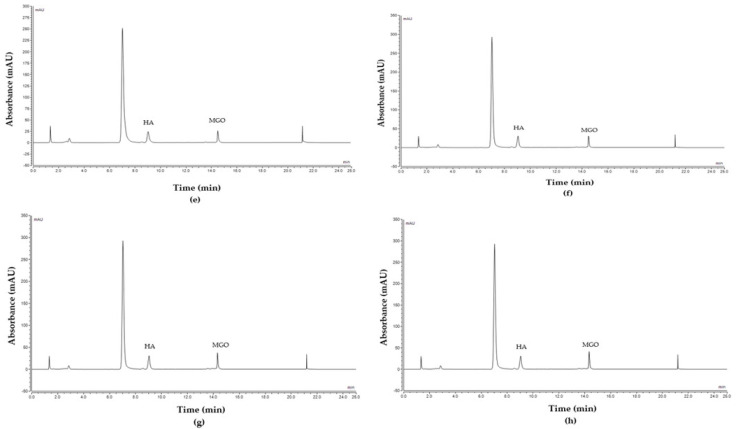
Peak profile of MGO released from the commercial ‘Product E’ at different time points: (**a**) 15 min, (**b**) 30 min, (**c**) 1 h, (**d**) 3 h, (**e**) 6 h, (**f**) 9 h, (**g**) 12 h and (**h**) Baseline.

Figure 4 shows the chromatograms obtained from the commercial ‘Product E’ collected during the course of the release study. No interference from formulation excipients in the analysis could be noted. The chromatograms of the other investigated formulations as well as the artificial and the pure Manuka honey are shown in the Supplementary File (Figures S1–S6).

**Figure 5:**
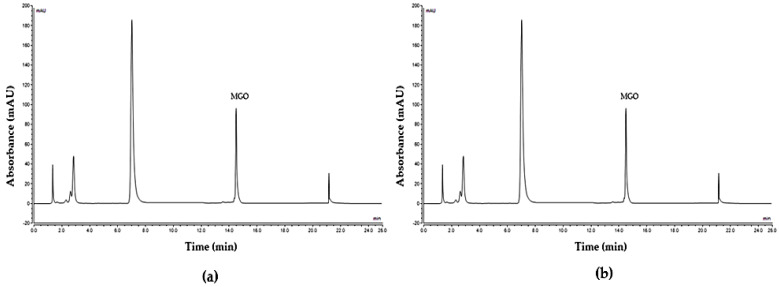
Peak profile of MGO solution (0.6 mg/mL) at RT 14.49 (**a**) Baseline (**b**) Released at 20 min. Buffer control gave additional peaks.

The pure MGO solution showed 100% release at 20 min. This is evident from Figure 5a, which shows the peak profile of pure MGO solution (0.6 mg/mL) with a peak area of 17.35 mAU and Figure 5b, which produced the same peak area for the sample collected after 20 min in the release study. The baseline MGO value of all commercial products and the two honeys (artificial and Manuka) were determined to quantify the MGO amount present in the samples at the start of the release experiment. This baseline value was used to calculate the release rate of MGO with the release of MGO at different time points expressed as % of the MGO baseline value in the respective samples.

**Figure 6:**
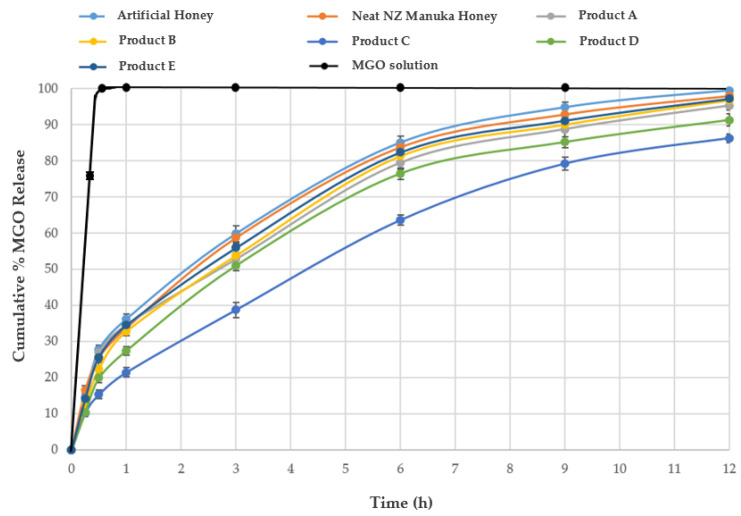
Cumulative % MGO release from pure MGO solution, five commercial products, spiked artificial honey and pure NZ Manuka honey at different time points.

The cumulative % MGO release is shown in Table 1 and Figure 6. The spiked artificial honey showed the highest percentage of MGO release (99.49%) over the course of 12 h whereas commercial ‘Product C’ displayed the lowest release (86.30%). All commercial products were fully dissolved in deionised water except product C and product D. It was also noted that more fine particles were present in product C compared to product D after dissolving the samples in deionised water, which might explain the difference in release observed for the two products (Figure 6). Dissolution rates (i.e., time taken to release 25%, 50%, and 75% of the baseline MGO content) for product C were also slower than corresponding rates observed for all other formulations and the two honeys (Table 2). There was no statistically significant difference (*p* = 0.985) in MGO release rate between the honeys and the formulations (except for product C).

## 3. Discussion

In vitro drug release/dissolution studies are considered to be an important indicator of product performance and quality. While these studies are common practice for conventional pharmaceutical dosage forms (i.e., tablets, capsules) and formulations with a limited number of active pharmaceutical ingredients, they should also be carried out for formulations that incorporate more complex natural products, such as honey [23,24].

As most honey-based products are designed for topical applications, the release of active components from the matrix is crucial [15,25,26,27]. It is therefore of interest to quantify MGO content in medicinal honeys as well as honey-based medicinal products and to monitor its release, as this is expected to reflect their therapeutic efficacy given MGO’s major role in the antibacterial activity of non-peroxide honeys. The MGO baseline value was determined at the start of the release study and taken to be the total MGO content in the samples for the determination of their MGO release profiles. It is interesting to note the significant variations in baseline MGO content (156–779 mg/kg) across the investigated formulations despite similarly high honey loading (80–99%). This illustrates that Manuka honeys have inherently different MGO levels and that this greatly influences the presence of MGO as a major antibacterial component in these formulations. Only one product (Product B) stipulated MGO content, and its baseline MGO concentration (779 mg/kg) was found to be very close to its declared content (800 mg/kg).

Next to variations in initial MGO content, the possible impact of other components in the honey matrix (e.g., sugars and non-sugar constituents) as well as of excipients in the honey-based formulations on the release of MGO also needs to be considered. In this study, it was found that the total release of MGO was not impacted by the honey’s non-sugar components as the % MGO release from artificial honey and pure Manuka honey over a period of 12 h was found to be 99.49 and 98.05%, respectively, and no statistically significant difference between the samples could be noted (*p* = 0.859). However, considering MGO’s prolonged release over the course of several hours from the two honey samples, it can be concluded that the honey’s sugar matrix creates an environment for the slow release. This can be seen in a comparative analysis with the release pattern of the MGO solution in the Franz diffusion cell, which fully transferred into the receptor compartment within 20 min. On the other hand, in the case of honey-based commercial products, the influence of excipients also needs to be taken into consideration. The excipients in the five commercial products tested in this study demonstrate a mixed influence on the release pattern of MGO (Table 1 and Figure 6). It was noticed that commercial Product A, B, and E displayed more than 95% MGO release over 12 h, presenting similar release patterns and no statistically significant difference in their final % MGO release (*p* = 0.798). However, the release of MGO was lower from Product C (86.30%) and also Product D (91.34%). These two products displayed a statistically significant (*p* < 0.0001) difference in the total percentage release of MGO compared to the other commercial products.

A potential explanation for the lower level of MGO release from these formulations might be related to the presence of natural oils and waxes as excipients in Product C and also Product D. These two products did not fully dissolve in water, as indicated by the presence of fine particles. Moreover, oils and waxes can adversely affect product wetting, which is a pre-requisite to water ingression into and subsequent release of MGO from the products. Furthermore, the amount of honey present in the formulation might also influence the rate of release with a higher excipient content, and thus, potentially also more hydrophobic excipients being present in formulations with lower honey content. These excipients might clog the pores of the dialysis membrane used in the Franz cell, which in turn might impact on the overall MGO release and also lead to a more delayed release pattern.

An essential factor influencing the outcome of localised drug delivery is the release rate of the active ingredient from the formulation matrix. When employed for wound healing, a fraction of active ingredients might be deactivated during its passage to the target location through contact with pro-inflammatory cytokines found within dead tissue [28,29]. Thus, the controlled release of MGO observed in this study might be advantageous for wound healing.

## 4. Materials and Methods

### 4.1. Samples

Five commercial products (Table 3) containing Manuka honey as the active ingredient were purchased from pharmacies and veterinary product suppliers in Australia. Most of the commercial products have a common application such as the treatment of various types of wounds, cuts, and burns. Apart from these, ‘Product E’ is marketed for the treatment of dry eye symptoms such as sore, irritated eyes and eyelids.

### 4.2. Chemicals and Reagents

Water filtered using 0.20 μm pore sized filters was used in all analyses. HPLC-grade acetonitrile (ACN) was obtained from RCI Labscan, Bangkok, Thailand. Hydroxyacetone (HA) (90%) and methylglyoxal (MGO) solution (40% *w*/*w* in water) were obtained from Sigma-Aldrich, Castle Hill, New South Wales, Australia. o-(2,3,4,5,6-Pentafluorobenzyl) hydroxylamine hydrochloride (PFBHA) (99%) was obtained from Alfa Aesar, Gymea, NSW, Australia. Spectra/Por^®^ Dialysis Membrane (MWCO: 3500) was sourced from Repligen, Waltham, MA, USA. NaCl and KCl were purchased from Chem Supply Pty Ltd., South Australia, and Na_2_HPO_4_ and KH_2_PO_4_ were obtained from Ajax Finechem, New South Wales, Australia.

### 4.3. In Vitro Release of MGO

All experiments were conducted in three independent vertical Franz-type diffusion cells (Scientific Equipment Manufacturers (S.E.M) (SA) Pty. Ltd., Magill, Australia) using 5 mL of phosphate-buffered saline (PBS) with pH 7.4 in the receptor compartment and a diffusion area of 0.78 cm^2^. Prior to the experiment, the dialysis membrane (in vitro mimic for skin) was cut into pieces of 4.5 cm^2^, incubated in PBS for 15 min, and mounted between the donor and the receptor chambers of the Franz cells, which were maintained at 37 °C using a water bath. The receptor chambers were filled with 5 mL sonicated PBS and stirred continuously with a magnetic bar (stirring speed set to ‘high’ mode). After spreading triplicate samples of 200 mg of pure honey and different honey formulations over the membrane surface areas, 300 μL samples were withdrawn from the receptor chambers at the following times: 15 min, 30 min, 1 h, 3 h, 6 h, 9 h, and 12 h. The samples became liquefied as they took up water over the 12 h study period. Moreover, 200 µL pure MGO solution (0.6 mg/mL) was also applied in triplicate onto the membrane as a control experiment. After each sample withdrawal, the volume removed from the receptor chamber was replaced with the same volume of fresh PBS solution. In the case of pure MGO solution, more frequent sampling was executed (every 10 min).

### 4.4. HPLC Analysis of Released MGO

The amount of MGO released from the collected samples was determined following a method described by Pappalardo et al., 2016 with minor modifications [22].

#### 4.4.1. HPLC Conditions

Analyses were performed on a Hewlett Packard Series 1100 Pump and Auto-sampler with a diode array detector (λ = 263 nm) (Agilent Technologies Australia Pty. Ltd., Mulgrave, Victoria, Australia). HPLC separations were performed on a Gemini NX-C18 column (150 × 4.6 mm, 5 μm particle size) obtained from Phenomenex, Lane Cove, NSW, Australia, 2066. The flow rate was set at 1.0 mL/min and the sample injection volume was 20 μL. Mobile phase A was 100% ACN and mobile phase B was 100% water. The following 25.0 min gradient elution was employed: A:B 37:63 (isocratic for 2.5 min), graded to 65:35 (over 8.0 min), graded to 100:0 (over 1.0 min), 100:0 (isocratic for 7.0 min), graded to 37:63 (over 1.0 min), 37:63 (isocratic for 5.5 min). Sample detection was carried out at 263 nm.

#### 4.4.2. Preparation of PFBHA Derivatisation Solution

PFBHA solution (19.8 mg/mL) was freshly prepared each time in 0.1 M citrate buffer and adjusted to pH 4.0 using 4 M NaOH.

#### 4.4.3. Preparation of HA Internal Standard Solution

HA internal standard solution was prepared by dissolving HA in water to a concentration of 3 mg/mL.

#### 4.4.4. Preparation of Artificial Honey with Known MGO Content

Artificial honey was prepared as reported previously [23] by dissolving 1.5 g sucrose, 7.5 g maltose, 40.5 g fructose, and 33.5 g glucose in 17 mL of sterile distilled water. Then, MGO solution (Sigma Aldrich, St. Louis, MO, USA) was added to the artificial honey at a concentration of 500 mg/kg to generate a ‘MGO 500’ artificial honey.

#### 4.4.5. Preparation of Standards

MGO standards were prepared in six different test tubes by adding first 0, 50, 100, 150, 200, 250 μL of a 0.6 mg/mL aqueous MGO stock solution, respectively, followed by HA standard solution (250 μL). The test tubes were thoroughly mixed and allowed to stand for 1 h to ensure complete mixing. PFBHA derivatising solution (1500 μL) was then added to each test tube. The resulting solutions were thoroughly mixed using a vortex mixer (MX-S, DLAB Scientific Co., Ltd., Beijing, China) and allowed to stand for a further 1 h for complete derivatisation. Next, ACN (6 mL) was added to each test tube and thoroughly mixed until all crystals dissolved and the solution turned clear. Water was added to each test tube to adjust the final volume to 10 mL and the solutions were again thoroughly mixed. A 1 mL aliquot of each sample was then placed in HPLC vials for analysis. The peak area ratios of MGO:HA were plotted against the known mass of MGO by linear regression to produce a MGO standard curve. The obtained calibration curve was then used to assess the MGO content of artificial honey spiked with a known amount of MGO, pure NZ Manuka honey, and also of different commercial honey-based formulations as well as the collected samples from the release study.

#### 4.4.6. Sample Preparation

One gram of each commercial product, artificial honey, and pure NZ Manuka honey was separately dissolved in deionised water to a concentration of 0.5 g/mL. Volumes of 250 µL of these solutions were transferred into test tubes followed by the addition of HA standard (250 μL). The resulting solutions were thoroughly mixed using a vortex mixer (MX-S, DLAB Scientific Co., Ltd.) and allowed to stand for 1 h. PFBHA derivatising solution (1500 μL) was then added and the solutions were again thoroughly mixed and allowed to stand for a further 1 h. ACN (6 mL) was added to each test tube and mixed until all crystals dissolved and the solution turned clear. Water was added to each test tube to bring the final volume to 10 mL and the test tubes were again thoroughly mixed. Aliquots (1 mL) of each sample were then placed in HPLC vials for analysis. All the commercial honey products were observed to completely dissolve in water except ‘Product C’ and ‘Product D’, which retained some small undissolved particles.

Volumes of 250 µL of the samples collected at different time points during the release study were transferred into test tubes, followed by the addition of HA internal standard, PFBHA derivatising solution, ACN, and water as described for the commercial samples and honeys.

### 4.5. Statistical Analysis

Graphpad Prism 9.4.1 (GraphPad Software, San Diego, CA, USA) was used for the analysis of variance (ANOVA) in order to determine whether there was a significant difference in the total amount of MGO release of different products including artificial and pure NZ Manuka honey. Moreover, Tukey’s post hoc comparisons were used to identify differences between the groups (*p* < 0.05).

## 5. Conclusions

This study investigated the release pattern of MGO from five honey-based (*Lepto-spermum* spp.) commercial products and the honey matrix itself. The findings provide a better understanding of the nature of MGO release from these samples. More than 90% of the initial MGO content was released from most investigated samples over a period of 12 h, with approximately 30% being released within the first hour of application. Overall, a slow and time-dependent release pattern for MGO was noticed from the honey matrix as well as from all investigated commercial products. While the honey matrix itself does not seem to have an influence on the total amount of MGO that is released, it was found that it impacts the rate of release, in addition to the potential impact of formulation excipients on MGO passage through the Franz cell’s diffusion membrane.

## Figures and Tables

**Table 1:** MGO Release Data (*n* = 3, data represents mean ± SD).

Sample *	MGO at Baseline	% MGO of Baseline Released at Different Time Points (h)						
		0.25	0.5	1	3	6	9	12
MGO solution	0.6 (mg/mL)	75.01 ± 0.90	100.00 ± 0.0	100.00 ± 0.0	100.00 ± 0.0	100.00 ± 0.0	100.00 ± 0.0	100.00 ± 0.0
Artificial Honey	500.11 ± 1.55 (mg/kg)	14.69 ± 0.94	27.81 ± 1.30	36.27 ± 1.40	59.89 ± 2.20	85.08 ± 1.80	94.81 ± 1.50	99.49 ± 1.90
Pure NZ Manuka Honey	348.53 ± 1.84 (mg/kg)	16.60 ± 1.20	25.65 ± 0.98	33.87 ± 1.60	58.82 ± 1.90	83.88 ± 1.60	92.94 ± 1.40	98.05 ± 1.30
Product A	252.55 ± 1.84 (mg/kg)	13.28 ± 0.95	26.95 ± 1.70	34.67 ± 1.40	52.81 ± 1.31	79.49 ± 1.91	88.74 ± 2.10	95.26 ± 1.20
Product B	779.42 ± 1.34 (mg/kg)	12.33 ± 1.40	22.49 ± 1.80	32.87 ± 1.20	53.86 ± 1.50	81.41 ± 1.80	90.11 ± 1.90	96.85 ± 1.40
Product C	605.45 ± 1.54 (mg/kg)	10.25 ± 1.10	15.37 ± 1.20	21.50 ± 1.30	38.80 ± 2.10	63.63 ± 1.40	79.24 ± 1.80	86.30 ± 1.20
Product D	700.14 ± 1.68 (mg/kg)	10.45 ± 1.20	19.97 ± 1.40	27.53 ± 1.20	51.06 ± 1.50	76.54 ± 1.60	85.23 ± 1.50	91.34 ± 1.70
Product E	156.03 ± 1.75 (mg/kg)	14.25 ± 1.30	25.72 ± 1.40	34.54 ± 1.60	55.95 ± 1.30	82.36 ± 1.10	91.13 ± 1.50	97.18 ± 1.40

* Commercial products are termed as ‘A, B, C, D and E’ to avoid potential conflicts of interest.

**Table 2:** MGO Release rate (*n* = 3, data represents mean ± SD).

Sample	Time (h) Required to Release 25, 50 and 75% MGO		
	T25%	T50%	T75%
MGO solution	0.083 ± 1.10	0.17 ± 1.20	0.25 ± 1.10
Artificial Honey	0.44 ± 0.94	2.09 ± 1.3	4.63 ± 1.43
Pure NZ Manuka Honey	0.49 ± 1.21	2.24 ± 0.98	4.75 ± 1.62
Product A	0.45 ± 0.95	2.69 ± 1.72	5.38 ± 1.41
Product B	0.56 ± 1.42	2.61 ± 1.81	5.13 ± 1.22
Product C	1.63 ± 1.10	4.26 ± 1.23	8.0 ± 1.34
Product D	0.78 ± 1.20	2.88 ± 1.42	5.75 ± 1.21
Product E	0.48 ± 1.30	2.38 ± 1.41	5.0 ± 1.61

**Table 3:** List of commercial products.

Product Name *	Product Type	Manuka Honey (%)	Stated MGO Content (mg/kg)	Listed Excipients	Claimed Application
Product A	Gel	80	Not stated	Sweet almond oil (*Prunus amygdalus*)	Contact layer for wounds such as venous ulcers, pressure ulcers (I–IV), diabetic Ulcers, 1st and 2nd degree burns, surgical wounds, donor and recipient graft sites, sloughy, malodorous wounds, general First Aid
Product B	Gel	99	800	Allantoin, propylene glycol	Burns, acute and chronic wounds, venous and arterial leg ulcers, diabetic, lower limb/foot ulcers, pressure sores, minor infection of postoperative wounds
Product C	Gel	80	Not stated	Natural oils and waxes	Non-healing and chronic wounds, traumatic, acute and surgical wounds, malodorous and sloughy wounds, burns and as a general first aid
Product D	Gel	80	Not stated	Natural wax and oils	Diabetic foot ulcers, leg ulcers, pressure ulcers/ sores, 1st and 2nd degree partial thickness burns, donor sites, and traumatic and surgical wounds
Product E	Gel	98	Not stated	Glycerol, Gum (*Acacia senegal*)	Dry eye symptoms such as sore, irritated eyes and eyelids. Assist the surface health of the eye by creating a microenvironment that supports healing and prevents further damage

* Commercial products are termed as ‘A, B, C, D and E’ to avoid potential conflicts of interest.

## Data Availability

Not applicable.

## Supplementary Materials

The following supporting information can be downloaded at: https://www.mdpi.com/article/10.3390/molecules28062858/s1, Figure S1: Peak profile of MGO released from spiked artificial honey at different time points: (a) 15 min, (b) 30 min, (c) 1 h, (d) 3 h, (e) 6 h, (f) 9 h, (g) 12 h and (h) Baseline; Figure S2: Peak profile of MGO released from pure NZ Manuka honey at different time points: (a) 15 min, (b) 30 min, (c) 1 h, (d) 3 h, (e) 6 h, (f) 9 h, (g) 12 h and (h) Baseline; Figure S3: Peak profile of MGO released from commercial ‘Product A’ at different time points: (a) 15 min, (b) 30 min, (c) 1 h, (d) 3 h, (e) 6 h, (f) 9 h, (g) 12 h and (h) Baseline; Figure S4: Peak profile of MGO released from commercial ‘Product B’ at different time points: (a) 15 min, (b) 30 min, (c) 1 h, (d) 3 h, (e) 6 h, (f) 9 h, (g) 12 h and (h) Baseline; Figure S5: Peak profile of MGO released from commercial ‘Product C’ at different time points: (a) 15 min, (b) 30 min, (c) 1 h, (d) 3 h, (e) 6 h, (f) 9 h, (g) 12 h and (h) Baseline; Figure S6: Peak profile of MGO released from commercial ‘Product D’ at different time points: (a) 15 min, (b) 30 min, (c) 1 h, (d) 3 h, (e) 6 h, (f) 9 h, (g) 12 h and (h) Baseline.
