# Supplementary material for: Monitoring the Release of Methylglyoxal (MGO) from Honey and Honey-Based Formulations
Source: Molecules. 2023 Mar 22;28(6):2858. doi: 10.3390/molecules28062858 (PMC10051060; doi:10.3390/molecules28062858)
Supplement: Supplementary file 1 [file molecules-28-02858-s001.zip › molecules-2259408-supplementary.pdf]

Supplementary Material

Monitoring the Release of Methylglyoxal (MGO) from Honey and Honey-Based Formulations

Md Lokman Hossain [1], Lee Yong Lim [1], Katherine Hammer [2,3], Dhanushka Hettiarachchi [1] and Cornelia Locher [1,3,*]

[1] Division of Pharmacy, School of Allied Health, University of Western Australia, Crawley 6009, Australia
[2] School of Biomedical Sciences, University of Western Australia, Crawley 6009, Australia
[3] Cooperative Research Centre for Honey Bee Products Limited, 128 Yanchep Beach Road, Perth 6035, Australia
* Correspondence: connie.locher@uwa.edu.au

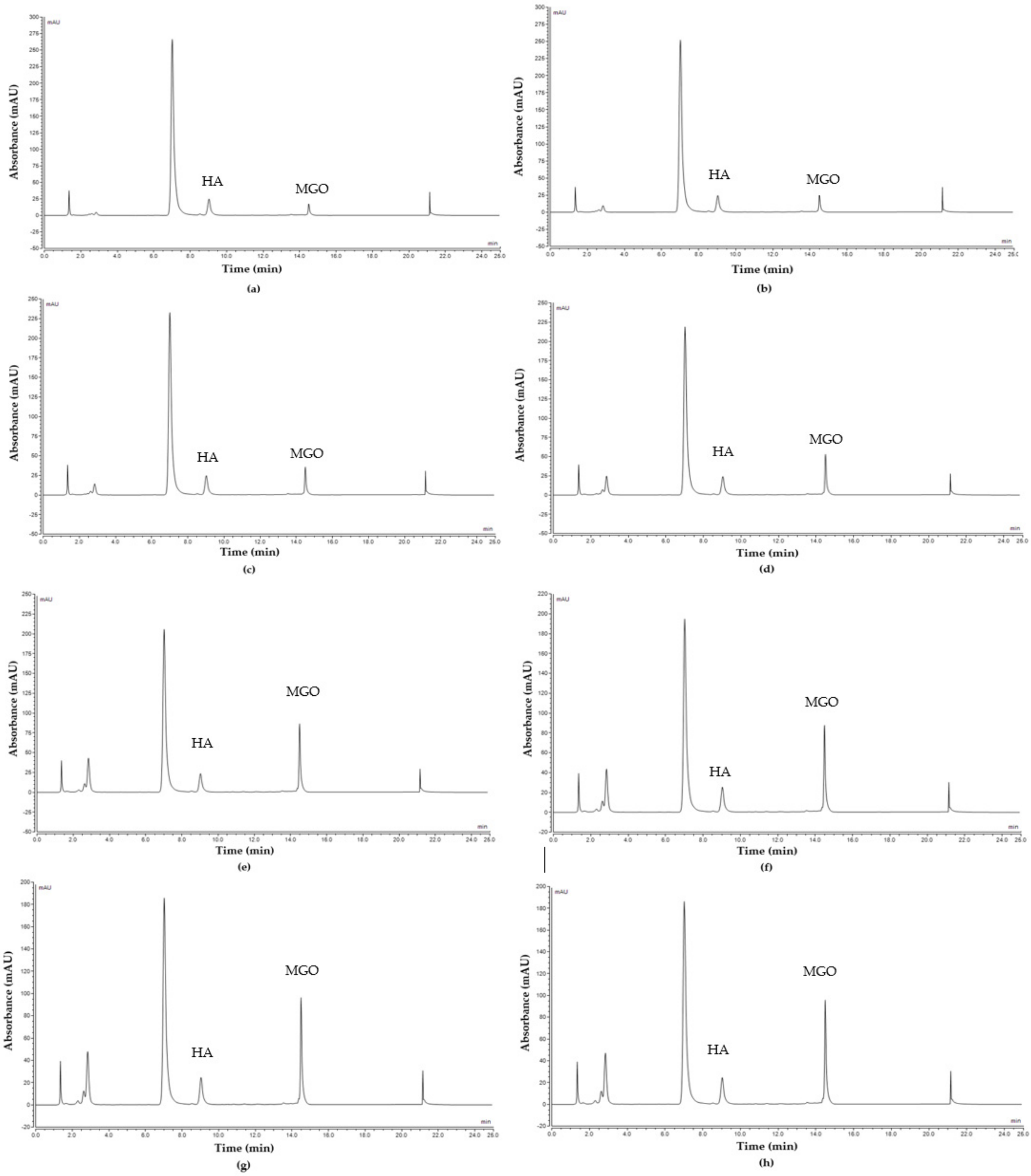

Figure S1. Peak profile of MGO released from spiked artificial honey at different time points: (**a**) 15 min, (**b**) 30 min, (**c**) 1 h, (**d**) 3 h, (**e**) 6 h, (**f**) 9 h, (**g**) 12 h and (**h**) Baseline.

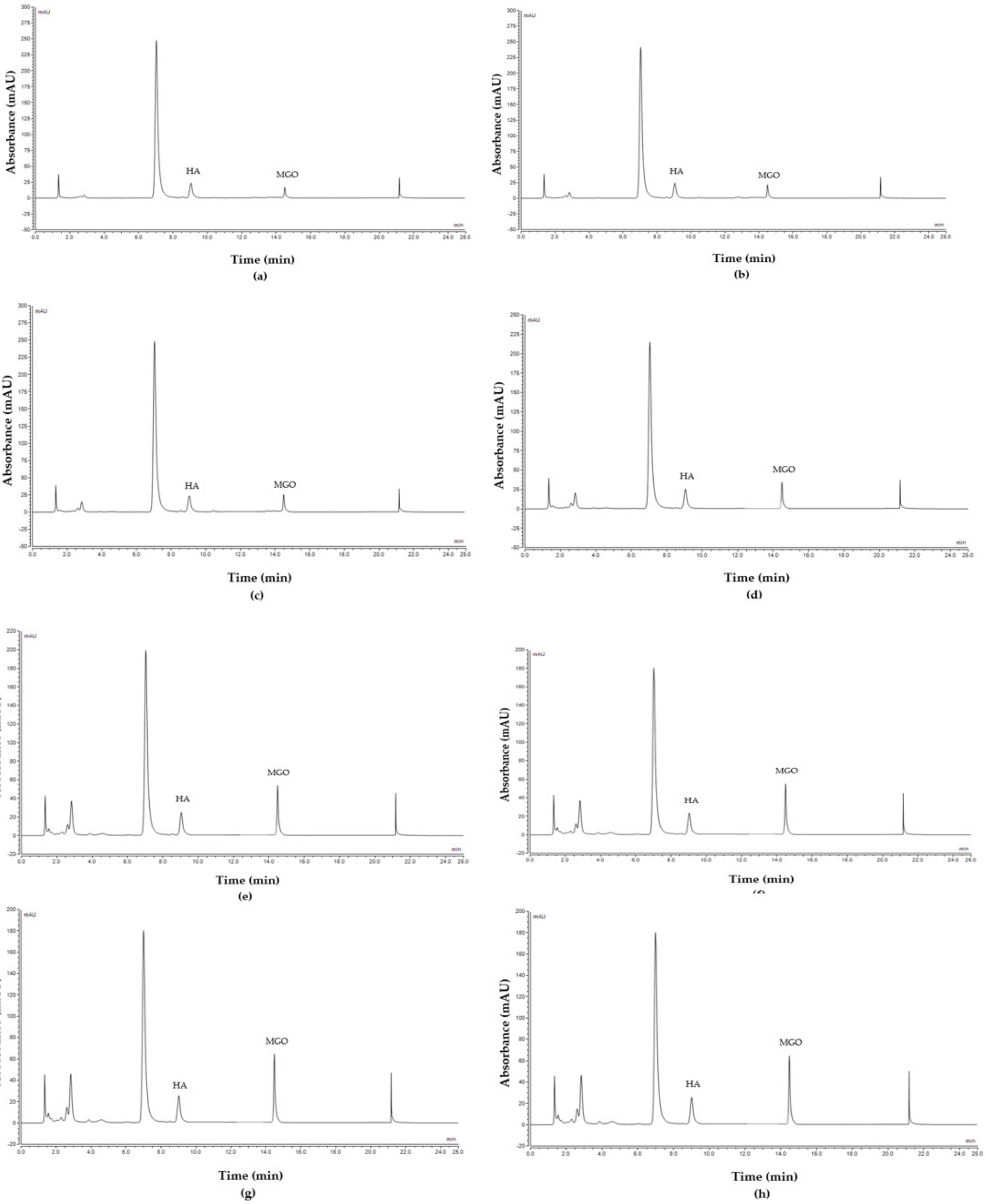

Figure S2. Peak profile of MGO released from Pure NZ Manuka honey at different time points: (**a**) 15 min, (**b**) 30 min, (**c**) 1 h, (**d**) 3 h, (**e**) 6 h, (**f**) 9 h, (**g**) 12 h and (**h**) Baseline.

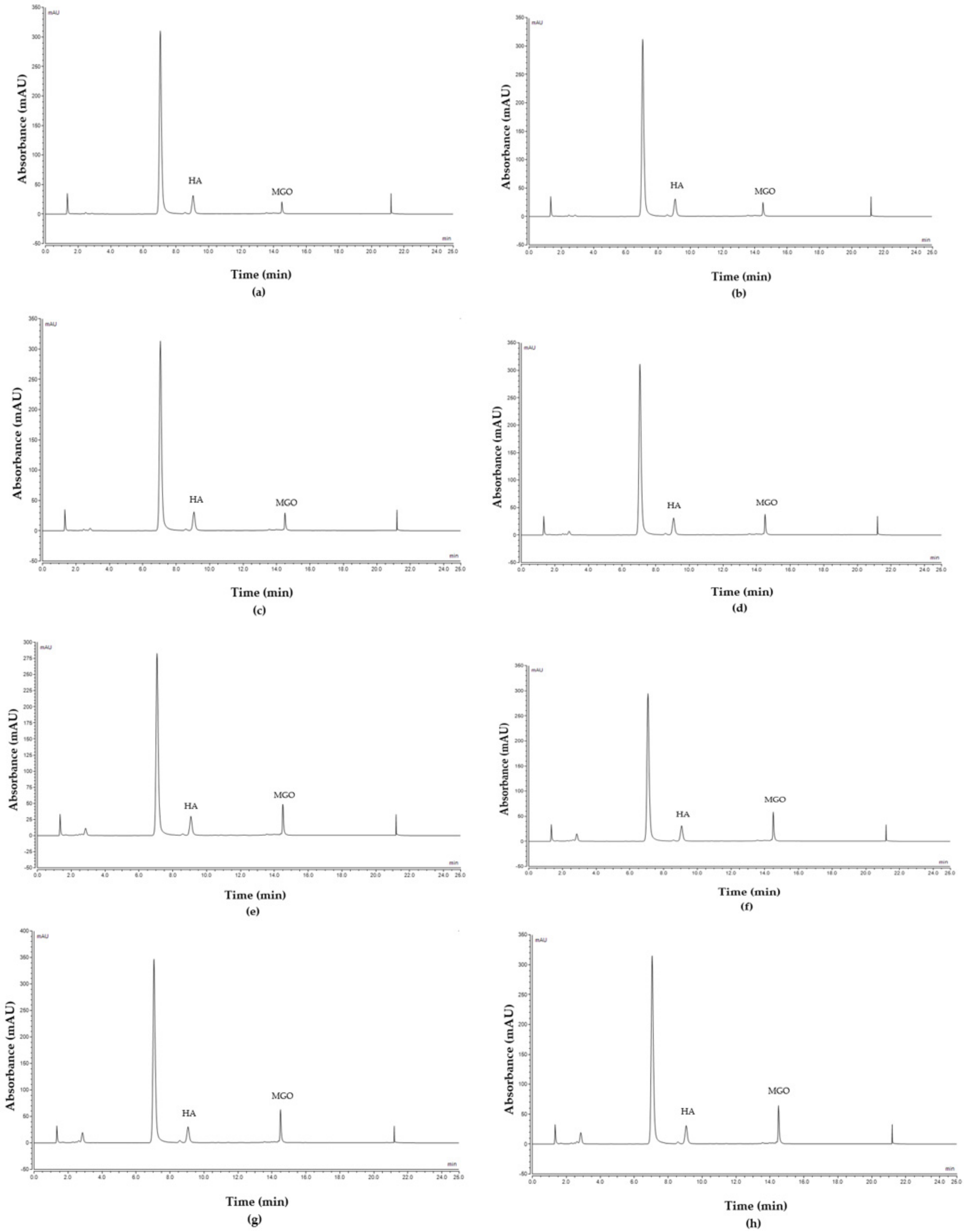

Figure S3. Peak profile of MGO released from commercial 'Product A' at different time points: (**a**) 15 min, (**b**) 30 min, (**c**) 1 h, (**d**) 3 h, (**e**) 6 h, (**f**) 9 h, (**g**) 12 h and (**h**) Baseline.

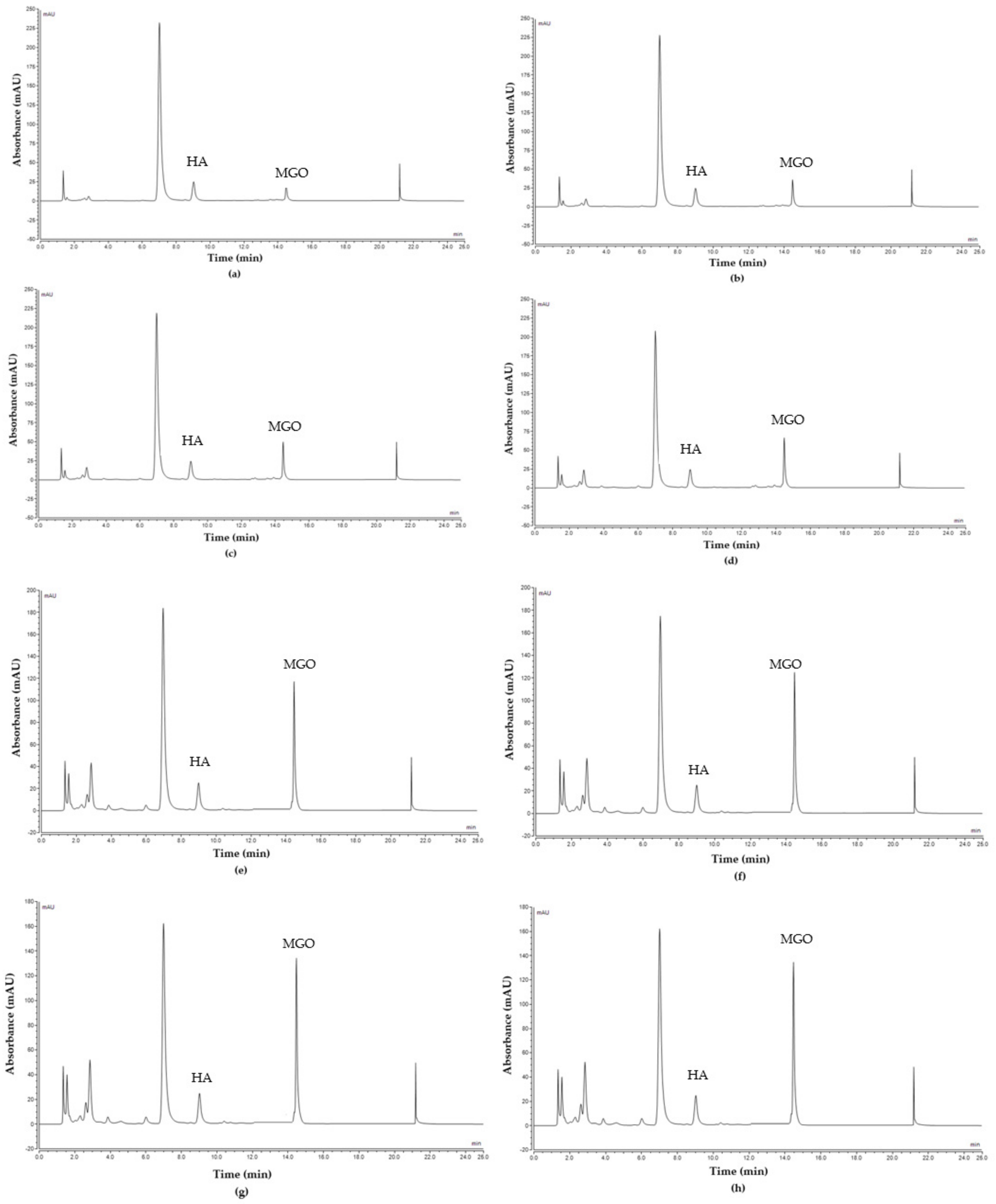

Figure S4. Peak profile of MGO released from commercial 'Product B' at different time points: (**a**) 15 min, (**b**) 30 min, (**c**) 1 h, (**d**) 3 h, (**e**) 6 h, (**f**) 9 h, (**g**) 12 h and (**h**) Baseline.

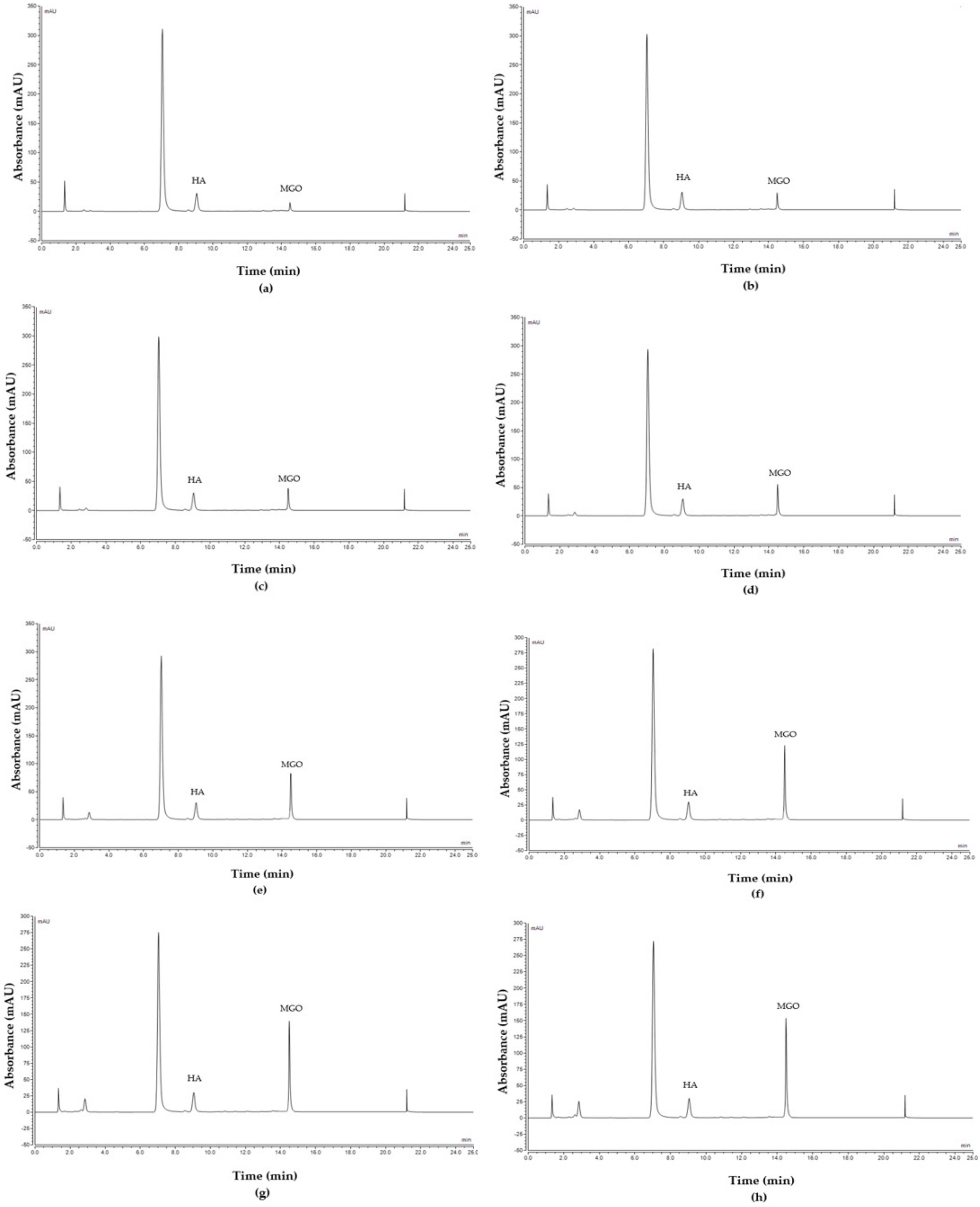

Figure S5. Peak profile of MGO released from commercial 'Product C' at different time points: (**a**) 15 min, (**b**) 30 min, (**c**) 1 h, (**d**) 3 h, (**e**) 6 h, (**f**) 9 h, (**g**) 12 h and (**h**) Baseline.

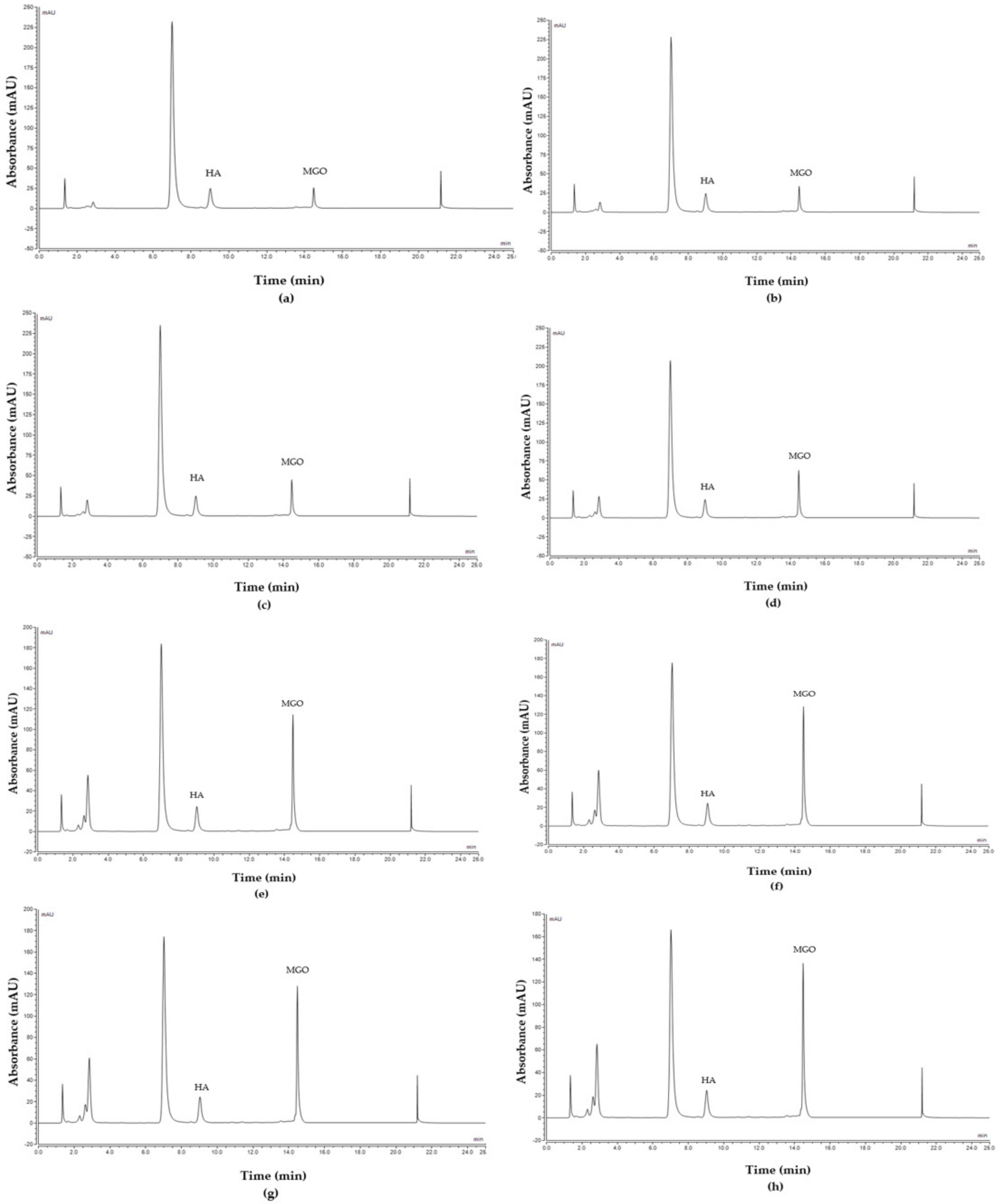

Figure S6. Peak profile of MGO released from commercial 'Product D' at different time points: (**a**) 15 min, (**b**) 30 min, (**c**) 1 h, (**d**) 3 h, (**e**) 6 h, (**f**) 9 h, (**g**) 12 h and (**h**) Baseline.
